# Evaluating the Quality and Reliability of YouTube Videos Providing Nutritional Recommendations for Irritable Bowel Syndrome

**DOI:** 10.1111/jhn.70088

**Published:** 2025-07-03

**Authors:** Eda Başmısırlı, Merve Kip, Hande Altun, Neriman İnanç

**Affiliations:** ^1^ Department of Nutrition and Dietetics, Faculty of Health Sciences Nuh Naci Yazgan University Kayseri Turkey

**Keywords:** accuracy, dietary advice, irritable bowel syndrome, Nutrition Scoring System, YouTube

## Abstract

**Purpose:**

This study aimed to evaluate YouTube videos providing nutritional recommendations for irritable bowel syndrome (IBS) in terms of validity, quality, accuracy and reliability.

**Methods:**

In December 2023, we searched for relevant videos on YouTube using three search terms related to IBS in Turkish. Two independent researchers analysed the content of 64 videos that met the inclusion criteria. Reliability and quality were determined using the m‐DISCERN criteria, the Global Quality Scale (GQS), the *Journal of American Medical Association* (JAMA) system, the Video Information and Quality Index (VIQI), and the IBS Nutition Scoring System (INSS).

**Results:**

The majority of these videos (%67.2) are produced by gastroenterologists, 15.6% by dietitians, and 17.2% by other individuals. The number of views and likes on videos by other individuals was higher compared to the videos of gastroenterologists (*p* < 0.05). In the comparison across the three groups, no statistically significant differences were found in the mean INSS (*p* = 0.287) and JAMA scores (*p* = 0.783). However, significant differences were observed in the mean interaction index (*p* = 0.029), Video Power Index (VPI) (*p* = 0.006), m‐DISCERN (*p* < 0.001), GQS (*p* = 0.002), and VIQI (*p* < 0.001) scores. GQS scores demonstrated strong positive correlations with both INSS and VIQI scores (*r* = 0.6528, *r* = 0.6174, respectively), while showing a moderate correlation with m‐DISCERN scores (*r* = 0.531, *p* < 0.001).

**Conclusion:**

Our study shows that users prioritise popularity over reliability when seeking nutritional information. Health professionals should create engaging content to ensure accurate information stands out online.

## Summary

- Most of the videos (67.2%) were uploaded by gastroenterologists.
- Videos uploaded by other users received more views and likes despite having lower quality scores.
- GQS scores showed a strong correlation with INSS and VIQI, and a moderate correlation with m‐DISCERN scores.

## 1 Introduction

Irritable bowel syndrome (IBS) is a functional gastrointestinal disorder characterised by recurrent abdominal pain, bloating, irregular defecation frequency and change in bowel habits, negatively affecting quality of life [1]. The symptoms usually appear before the age of 50 [2]. According to the American College of Gastroenterology (ACG), at least 12% of the population worldwide suffers from IBS [3]. The prevalence of IBS in Turkey varies between 6.2% and 19.1% [4]. IBS is difficult to diagnose because there are no blood tests, exams, or procedures to definitively diagnose it. IBS evaluation; it is done by excluding diagnoses with similar symptoms, such as inflammatory bowel disease, motility disorders, and coeliac disease [5]. Diagnosis is made according to Rome IV criteria based on gastrointestinal symptoms [6]. The development of IBS is associated with various biological, psychosocial, and nutritional factors, in addition to gastrointestinal infections. Psychosocial and environmental influences, including education level, socioeconomic status, female gender, depression, anxiety, stress, sleep disturbances, and family history of IBS, may contribute to its risk. However, the frequent use of health services observed in IBS patients may reflect a response to symptoms rather than a direct risk factor for developing the condition. In addition, comorbidities such as diabetes mellitus, nutritional habits such as inadequate water consumption, inadequate fibre intake, and caffeine consumption may also play a role in the development of IBS. Among these factors, education level and socioeconomic status in particular may shape individuals' access to nutritional information and their nutritional habits. For example, individuals with low education levels may use online resources such as YouTube more frequently to obtain nutritional information rather than traditional health services. This is an important aspect of nutritional management in IBS [7, 8].

Nutrition‐based interventions play an important role in better understanding and managing IBS risk factors. Among the various nutritional interventions applied to alleviate IBS symptoms, the low Fermentable oligosaccharides, disaccharides, monosaccharides, and polyols (FODMAP) diet stands out. However, it should be noted that long‐term implementation of a low FODMAP diet carries some risks [9]. Therefore, the long‐term superiority of the low FODMAP diet over traditional IBS diets, such as those recommended by the National Institute for Health and Care Excellence (NICE), needs to be demonstrated [10]. Therefore, patients' symptoms should be carefully monitored and a personalised nutrition programme should be created [11].

There is currently no definitive nutritional treatment for IBS [12]. In addition, due to reasons such as long waiting times in the healthcare system, lack of trust in the healthcare system, and the fact that IBS is based on individual experience, individuals turn to different sources for information [13, 14]. YouTube is an open access video sharing platform used for this purpose, allowing users to access unlimited video content and upload videos [15]. YouTube is one of the most visited platforms thanks to its free access and 2 billion active users worldwide, 100 million daily viewers, more than 5 billion videos shared to date [16].

Individuals can choose YouTube to get nutrition advice because it is easy to access information and has verbal and auditory explanations [17]. However, there is no mandatory editing and peer review system for health videos uploaded to YouTube. Therefore, videos presented to users may lead to the spread of false or misleading information [18].

In recent years, various studies have been conducted on the relevance, accuracy, reliability and quality of health‐related videos on YouTube [19, 20, 21, 22]. In the literature, in the study of Gkikas et al., perceptions of foods in YouTube videos on the management of Inflammatory Bowel Disease (IBD) were classified as positive, negative and neutral. Accordingly, foods that were claimed to be beneficial or harmful were determined [23]. Similarly, other studies have examined the credibility of nutritional information shared on YouTube [24, 25]. Various tools such as Global Quality Scale (GQS), DISCERN and The Journal of American Medical Association (JAMA) were used to evaluate videos in the studies. However, studies has found that YouTube videos are of low quality on various topics such as cancer, reflux, fibromyalgia and dental diseases and cannot be used as a source of information [19, 20, 21, 22].

Therefore, the aim of this study was to evaluate the validity, quality, accuracy, and reliability of YouTube videos that provide nutritional recommendations for IBS. In the literature, there is only one YouTube study examining the relationship between IBS and nutrition, but there is no study on this in Turkey. For this reason, this study is the second in the literature and the first in Turkey.

## 2 Materials and Methods

On 5 December 2023, we searched YouTube for videos resulting from three search terms related to IBS and Nutritional Therapy. In Turkish; ‘İrritabl Bağırsak Sendromunda Beslenme’, ‘Huzursuz Bağırsak Sendromunda Beslenme’, and ‘Hassas Bağırsak Sendromunda Beslenme’. No restrictions were applied regarding the age group (e.g., adults or children), IBS subtype (e.g., IBS‐C, IBS‐D and IBS‐M), or target audience of the videos. Videos that provided any form of nutritional recommendations related to IBS were included in the study.

The evaluation of video content was carried out independently by two intern dietitians who were not involved as authors and were blinded to each other's assessments. Before starting the video evaluations, training on the tools used, such as INSS, VIQI, modified‐DISCERN, GQS and JAMA criteria, was provided to two intern dietitians by the researchers. Each intern dietitian assessed all included videos using five structured tools. To ensure the objectivity and consistency of the evaluations, we calculated the intraclass correlation coefficient (ICC) to assess inter‐rater reliability.

The search was carried out by deleting cookies and caching with Google Chrome, applying by default settings without filters. Thousands of videos were generated through each of the search terms, making it impossible to analyse all the videos. Therefore, first 100 videos generated for each search term (*n* = 300) were assessed for eligibility as in prior research [26].

The inclusion criteria were videos in the Turkish language with a duration of at least 1 min, and giving dietary advice for IBS. Videos were excluded if they were not related to IBS. Additionally, videos longer than 20 min were excluded, as they are less likely to be watched in full [27]. In case of duplicated videos, the first video was used for analysis, and subsequent videos were excluded. YouTube Shorts and ads were also excluded. After excluding duplicate videos (*n* = 138), videos that did not mention IBS (*n* = 79), videos shorter than 1 min or longer than 20 min (*n* = 19) the study was completed with 64 videos.

### 2.1 Video Characteristics

For each video, data was collected for the number of channel subscribers, video duration (minutes), time since upload (days), number of views, number of likes, number of dislikes, and number of comments. This data contributed to the calculation of metrics such as Interaction Index, and Video Power Index (VPI). The source of each YouTube video was categorised as ‘gastroenterologist,’ ‘dietitian,’ or ‘other’. Other professionals included chemists (*n* = 2), personal development specialists (*n* = 2), a psychologist (*n* = 1), patient advocates (*n* = 2), a physical therapy specialist (*n* = 1), a chiropractor (*n* = 1), a natural medicine practitioner (*n* = 1) and an individual with no specific title (*n* = 1). When available, the profiles of video posters were reviewed to assess any listed credentials. However, no in‐depth verification of the poster's identity or credentials was conducted beyond the information provided on their publicly accessible profile pages. Audience engagement data were collected on 5 December 2023. User interaction with the videos was recorded through likes, dislikes, views, and subscriber counts. The interaction index was calculated using the formula: Interaction index = ((number of likes − number of dislikes/total number of views) × 100%) [28, 29]. The view ratio = (number of views/number of days since upload) × 100%) and the Like Ratio = ((number of likes × 100)/(number of likes + number of dislikes)) × view ratio)/100)) used for calculation of Video Power Index ((like ratio × view ratio)/100) [30].

### 2.2 Quality, Reliability, Accuracy, and Validity of Videos

Quality: Refers to the overall presentation, structure, and clarity of the video content. Quality was evaluated using the Video Information and Quality Index (VIQI) and the Global Quality Scale (GQS).

Reliability: Refers to the trustworthiness and consistency of the information source. In this study, reliability was assessed using the modified‐DISCERN tool, which evaluates the reliability of health‐related video content. Additionally, to ensure consistency in the evaluation, all videos were independently assessed by two reviewers, and inter‐rater reliability was calculated using Cohen's kappa statistic.

Accuracy: Refers to the degree to which the information in the video aligns with current, evidence‐based clinical guidelines. Accuracy was assessed using the IBS Nutrition Scoring System (INSS) and the Video Information and Quality Index (VIQI).

Validity: Refers to the extent to which the tools used in the study measure what they are intended to measure. Validity was ensured by developing the INSS based on evidence‐based clinical guidelines and by using previously validated tools such as the GQS, JAMA and VIQI.

### 2.3 The Global Quality Score 5‐Point Scale (GQS)

The GQS was used to rate YouTube videos on their overall educational quality and information flow [31]. GQS evaluates videos on a 5‐point scale, with 1 being the lowest and 5 being the highest. Videos containing unhelpful, low‐quality, and incomplete information are rated 1 point, videos providing limited benefit, low quality, and limited information are rated 2 points, videos providing moderate benefit and discussing only some important information are rated 3 points, videos providing useful, high‐quality content and video flow, containing relevant information but not covering some topics are rated 4 points, and videos covering all information and excellent quality and flow, very useful for patients, receive 5 points [32].

### 2.4 Modified‐DISCERN

The reliability of videos was assessed using the m‐DISCERN tool [32]. This assessment tool consists of five yes (1) ‐ no (0) questions, and it has a scoring system ranging from 1 to 5, with the minimum possible score being 1 and the maximum score being 5. In terms of information reliability, a score of 1 or 2 points for the questions indicates a ‘poor’ level, 3 points indicate a ‘fair’ level, and a score of 4 or 5 points indicates a ‘good’ level [32].

### 2.5 Video Information and Quality Index (VIQI)

The VIQI Scale was used to assess the accuracy, flow, quality, and precision of the videos. The scale uses a 5‐point Likert scale, with 1 indicating poor quality and 5 indicating high quality, to evaluate video characteristics: information flow, accuracy, quality (one point each for use of still images, interview with individuals in the community, video captions, and a report summary), and precision (the coherence between the title and content). The score ranges from 5 to 20 [33, 34, 35].

### 2.6 The *Journal of American Medical Association* (JAMA)

The JAMA benchmark criteria, a system for assessing the validity of health‐related online resources, was four main criteria as follows: authorship, citation, explanation, and up‐to‐date status. Each criterion was graded on a scale of “0” or “1”. Total score ranging from 0 to 4, with a maximum score of 4 indicating the highest video reliability [36].

### 2.7 Alignment of the Videos With Guideline Recommendations

The m‐DISCERN, GQS, VIQI and JAMAS scoring systems, although frequently used in the literature, do not provide specific assessments of the IBS‐related nutrition videos tested in this study. To address this limitation and evaluate YouTube videos in a way that is more aligned with the objective of our study, we developed an IBS nutrition scoring system (INSS). To assess the accuracy and comprehensiveness of dietary recommendations for IBS, the videos were scored against information identified in three evidence‐based IBS dietary guidelines to form the basis of a compliance scoring system. These guidelines were 2021 updated ACG Clinical Guideline: Management of IBS [3], 2016 British Dietetic Association Systematic Review And Evidence‐Based Practice Guidelines for The Dietary Management of IBS in Adults [37], and 2017 IBS in Adults: Diagnosis and Management [38].

Based on these guidelines, 19 key dietary elements were identified and included in the scoring item (Supporting Information: Table 1). These elements covered recommendations such as the adoption of a low FODMAP diet, the use of soluble fibres, peppermint, probiotics, adjustments to meal frequency, hydration, caffeine and alcohol intake, as well as the restriction of fat, dairy, spicy foods, resistant starches and sorbitol. Recommendations specific to symptom subtypes (e.g., flaxseed for constipation, avoiding aloe vera, and the role of oats for bloating) were also included.

Each video was evaluated for its inclusion of these elements. A score of 1 point was given if the video presented the item accurately and in accordance with the guidelines, and 0 points if the item was not mentioned or was inaccurately presented. The method was developed to enable the analysis of videos.

### 2.8 Statistical Analysis

Statistical analyses were performed using the Statistical Package for the Social Sciences 22 (SPSS). Kolmogorov–Smirnov tests were used to assess the normality of continuous data. To determine whether the characteristics, reliability and quality of videos differ based on their presenter the Kruskal–Wallis test (no data found with normal distribution) and after Tamhane T2 post hoc test (Bonferroni correction was used to control the error rate due to multiple comparisons) was performed. Besides, to assess the associations between the quantifying variables, we applied Spearman's rank correlation.

We sought to establish at least moderate agreement on index and scoring systems by assessing the ICC for continuous measures (values between 0.50 and 0.75 indicate moderate reliabilityvalues, between 0.75 and 0.9 indicate good reliability, and values greater than 0.90 indicate excellent reliability [39]. ICC estimates and their 95% confident intervals were calculated based on a mean‐rating (*n* = 2), absolute‐agreement, two‐way mixed‐effects model [40].

## 3 Results

In the study, 64 YouTube videos that met the inclusion criteria were analysed. The majority of these videos (67.2%) are produced by gastroenterologists, 15.6% by dietitians, and 17.2% by other individuals. The average duration of the videos is 3.80 min, and the upload duration is 1195 days. The number of channel subscribers for other individuals (181.000) was statistically significantly higher compared to dietitians (4.745). The number of views and likes on videos by other individuals was higher compared to the videos of gastroenterologists (*p* < 0.05). Compared to the videos of other individuals and dietitians, the number of comments on gastroenterologists' videos is statistically significantly lower (*p* < 0.001). In the comparison across the three groups, no statistically significant differences were found in the mean INSS (*p* = 0.287) and JAMA scores (*p* = 0.783). However, significant differences were observed in the mean interaction index (*p* = 0.029), VPI (*p* = 0.006), m‐DISCERN (*p* < 0.001), GQS (*p* = 0.002) and VIQI (*p* < 0.001) scores. Post‐hoc analysis revealed that the differences in the m‐DISCERN, GQS, and VIQI scores were primarily attributable to the disparities between the videos uploaded by others and those uploaded by dietitians and gastroenterologists. The observed variations in interaction index scores were primarily between videos uploaded by dietitians and gastroenterologists (respectively 2.07, 0.72). Videos uploaded by other individuals demonstrated significantly higher VPI scores compared to those uploaded by gastroenterologists (Table 1).

**Table 1:** General information regarding YouTube™ videos and comparison of video parameters based on the source of upload.

Parameters	All (*n* = 64)	Dietitian (10)	Gastroenterologist (*n* = 43)	Other (*n* = 11)	Test statistic	*p* value*
	Median (Q1–Q3	Median (Q1–Q3)	Median (Q1–Q3)	Median (Q1–Q3)		
Channel subscribers	10.650 (2207–70.200)	4745 (1268.25–10175)^b^	12.800 (2200–54.200)^ab^	181.000 (6500–612,000)^a^	6.5946	**0.037**
Video duration (minutes)	3.80 (1.55–8.32)	4.66 (2.46–5.53)	3.32 (1.55–8.4)	6.14 (1.48–10.38)	0.7554	0.685
Time since upload (day)	1195 (884–2013)	989.5 (813–1166.5)	1350 (892–2053)	1073 (432–2399)	2.8623	0.239
Number of views	6667 (1318–229.999)	6224.5 (670–20997)^ab^	4342 (1106–13.583)^b^	12.503 (9522–123,000)^a^	7.1598	**0.028**
Number of likes	56.50 (12.25–169.00)	95.5 (14.25–215.25)^ab^	26 (8–117)^b^	100 (74–3300)^a^	8.6853	**0.013**
Number of dislikes	0 (0–8.5)	2.5 (0–10.25)	0 (0–4)	6 (0–91)	5.2152	0.074
Number of comments	3.00 (0.00–45.00)	27.5 (1.75–149)^a^	0 (0–14)^b^	115 (4–220)^a^	15.8321	**< 0.001**
Interaction index	0.83 (0.54–1.93)	2.07 (0.87–3.00)^a^	0.72 (0.52–1.54)^b^	1.77 (0.57–2.22)^ab^	7.051	**0.029**
VPI	507.31 (98.68–1,568.29)	518.06 (120.82–1754.26)^ab^	252.69 (78.73–1183.75)^b^	1526.45 (801.52–12106.99)^a^	10.3294	**0.006**
m‐DISCERN	3.00 (2.00–3.00)	3.00 (2,00–3.00)^a^	3.00 (2.00–3.00)^a^	2.00 (1.00–2.00)^b^	17.0045	**< 0.001**
GQS	3.00 (3.00‐3.37)	3.50 (3.00–4.00)^a^	3.00 (3.00–3.50)^a^	3.00 (2.00‐3.00)^b^	12.8623	**0.002**
INSS	1.00 (0.00‐4.00)	2.00 (1.00–7.50)	2.00 (0.00–3.00)	1.00 (0.00–4.00)	2.5578	0.287
VIQI	15.00 (14.00–16.00)	16.00 (15.88–16.13)^a^	15.00 (15.00–16.13)^a^	12.00 (11.00–14.00)^b^	20.4766	**< 0.001**
JAMA	6.00 (6.00–6.00)	6.00 (6.00–6.00)	6.00 (6.00–6.00)	6.00 (6.00–6.00)	0.4883	0.783

Abbreviations: GQS, Global Quality Scale; INSS, IBS Nutition Scoring System; JAMA, *Journal of American Medical Association*; VIQI, Video Information and Quality Index; VPI, Video Power Index.

The intraclass correlation coefficient (ICC) was utilised to assess the inter‐rater reliability between two evaluators based on scores assigned to video quality assessment scales. Results indicated that for all five scales, the ICC values reflected excellent reliability between evaluators (ICC > 0.90) (Table 2).

**Table 2:** Intraclass correlation coefficients for interrater reliability.

	Intraclass correlation	95% Confidence interval		*F* test with true value 0			
		Lower bound	Upper bound	Value	*df*1	*df*2	Sig
JAMA	0.947	0.913	0.968	18.755	63	63	< 0.001
m‐DISCERN	0.979	0.965	0.987	47.115	63	63	< 0.001
GQS	0.962	0.935	0.977	27.983	63	63	< 0.001
INSS	0.997	0.995	0.998	340.254	63	63	< 0.001
VIQI	0.988	0.980	0.993	88.980	63	63	< 0.001

*Note:* All estimates are from average measures. Single‐rating, absolute‐agreement, 2‐way random‐effects model.

Abbreviations: GQS, Global Quality Score; INSS, IBS Nutrition Scoring System; JAMA, *Journal of American Medical Association*; m‐DISCERN, modified DISCERN; VIQI, Video Information and Quality Index; VPI, Video Power Index.

Table 3 presents the correlations between the scores of the scales used in the analysis. Correlation analysis revealed a moderate positive correlation between interaction index scores and both INSS and GQS scores (*p* < 0.001). GQS scores demonstrated strong positive correlations with both INSS and VIQI scores (*r* = 0.6528, *r* = 0.6174, respectively), while showing a moderate correlation with m‐DISCERN scores (*r* = 0.531, *p* < 0.001). Furthermore, the VIQI score demonstrated a negetive correlation with the INSS score (*r* = ‐0.4006, *p* = 0.001). Additionally, the m‐DISCERN score was found to have a moderate positive correlation with VIQI (*p* < 0.001) and a low positive correlation with INSS score (*p* = 0.020). The strongest correlation was observed between view rates and VPI scores (*r* = 0.970, *p* < 0.001).

**Table 3:** Correlation matrix between variables.

*p r*	VPI	Interaction Index	View Ratio	INSS	VIQI	m‐DISCERN	JAMA	GQS
VPI	1	0.1494	**0.9700**	0.1089	−0.1591	−0.0803	0.0047	0.0959
Interaction index	0.239	1	0.0859	**0.5358**	0.1111	0.1062	0.045	**0.4514**
View ratio	**< 0.001** ***	0.500	1	0.0588	−0.1694	−0.0677	−0.0056	0.0387
INSS	0.392	**< 0.001** ***	0.644	1	**−0.4006**	**0.2899**	0.0735	**0.6528**
VIQI	0.209	0.382	0.181	**0.001** **	1	**0.439**	0.0909	**0.6174**
m‐DISCERN	0.528	0.403	0.595	**0.020** *	**< 0.001** ***	1	0.2376	**0.531**
JAMA	0.971	0.724	0.965	0.564	0.475	0.059	1	0.1831
GQS	0.451	**< 0.001** ***	0.762	**< 0.001** ***	**< 0.001** ***	**< 0.001** ***	0.148	1

*Note:* Spearman's Corralation.

Abbreviations: GQS, Global Quality Score; INSS, IBS Nutrition Scoring System; JAMA, *Journal of American Medical Association*; m‐DISCERN, modified DISCERN; VIQI, Video Information and Quality Index; VPI, Video Power Index.

* *p* < 0.05

** *p* < 0.01

*** *p* < 0.001.

## 4 Discussion

This study found that the quality and credibility of YouTube videos offering nutritional advice for individuals with IBS varied significantly. Content produced by healthcare professionals tended to be of higher quality, while videos uploaded by laypersons were more popular but lower quality. Videos uploaded by healthcare professionals also received less interaction. Additionally, no direct relationship was found between popularity and quality. These results highlight the need for healthcare professionals to develop more effective strategies to disseminate quality information to a wider audience.

The widespread use of social media has led to a significant increase in the sharing of health‐related content on platforms such as YouTube [40]. The ability of anyone to upload videos without the need for peer review raises concerns about the spread of misinformation, treatments that are not scientifically supported, and the generalisation of information that is beneficial to the individual [41]. As a result, assessing the quality and reliability of YouTube videos is crucial [42]. Consequently, the objective of this study was to evaluate the relevance, quality, accuracy, and reliability of YouTube videos offering nutritional recommendations for individuals with IBS.

In our study, the low FODMAP diet was prominent in the videos evaluated for reducing IBS symptoms. The low FODMAP diet is a nutritional model that includes foods low in fermentable oligosaccharides, disaccharides, monosaccharides, and polyols. High FODMAP fruits (apple, pear, peach, apricot and watermelon), vegetables (onion, garlic, cauliflower and peas), grains (bread, wheat, rye and barley), milk and dairy products (cheese, yogurt, cream and ice cream) improvement in symptoms may be seen by restricting the diet [43]. In the low‐FODMAP diet, high‐FODMAP foods are removed from the diet every 4–6 weeks. When complaints decrease, high‐FODMAP foods are added to the diet, one every day, and symptoms are monitored. Depending on the patient's response to foods, foods that increase symptoms are removed from the diet [44]. In our study, repeated nutritional recommendations were examined in 64 videos analysed using the INSS method. The most frequently suggested practice was the use of probiotics (*n* = 7). This was followed by recommendations to limit the consumption of dairy products and to restrict the intake of alcohol and/or carbonated beverages, each mentioned in six videos. Notably, none of the analysed videos included any recommendations regarding the use of aloe vera and the consumption of flaxseed, oats, high‐fibre and soluble fibre‐rich foods (Supporting Information: Figure S1).

YouTube is becoming increasingly popular among healthcare professionals due to its ease of use and accessibility [45]. The majority of the videos evaluated in our study (67.2%) were uploaded by gastroenterologists (Table 1). Other studies have also shown that most of the uploaded videos are uploaded by health professionals and health organisations [46, 47]. Our study demonstrates that non‐healthcare professionals attain the highest scores in terms of channel subscribers, video views, likes, and comments (*p* < 0.05, Table 1). This could be because non‐healthcare professionals tend to engage in personal experiences and visually engaging content that can attract a wider audience. Additionally, YouTube's algorithmic processes may prioritise content that generates more engagement, further increasing the reach of nonprofessional content creators. In addition, the VPI is used to evaluate the interaction and impact of videos with viewers and in our study it showed a very strong correlation with the view ratio score (*r* = 0.970, *p* < 0.001, Table 3). The observation that the VPI attained the highest score in groups other than gastroenterologists is further elucidated by the interaction index, which shows a significant difference (*p* = 0.006, Table 1). However, the interaction index score in videos uploaded by dietitians was higher than that of gastroenterologists (*p* = 0.029, Table 1). This shows that there is great interest in the videos uploaded by dietitians and that the content successfully reaches the target audience. Other studies have found that VPI is strongly correlated with viewing rates, videos produced by non‐experts have higher VPI values, and VPI is not directly linked to scientific quality, and similar results were obtained with our study [48, 49, 50, 51].

As YouTube's popularity increases, many people turn to YouTube to learn about their illness [52]. Therefore, in our study, GQS, m‐DISCERN, VIQI and JAMA scoring tools were used to evaluate the quality and reliability of the videos. In our study, it was observed that the m‐DISCERN, GQS and VIQI scores of the videos uploaded by dietitians were higher than other upload sources (*p* < 0.05, Table 1). In studies conducted in China, Turkey and Korea, respectively, examining videos on nutrition in inflammatory bowel disease, nutrition after bariatric surgery, and the effect of vitamin C on the common cold, it was found that the m‐DISCERN and GQS scores of videos uploaded by dietitians were higher [53, 54, 55]. The higher scores of dietitians can be explained by the fact that they prepare content based on scientific foundations thanks to the education they receive. These findings suggest that dietitians provide a higher standard of quality and play an important role in delivering reliable nutrition information to the public. However, it should not be overlooked that gastroenterologists also prepare quality content based on scientific foundations [53]. The relatively lower scores they received in video evaluations compared to other groups may be due to their greater focus on clinical practice.

The reliability of the scales assessing the quality of the videos was confirmed by ICCs (Table 2). In the correlation analysis, a very strong positive relationship was found between VPI and view rate (*r* = 0.970, *p* < 0.001, Table 3). This result supports other studies showing that the VPI value increases as the video viewing rate increases [56, 57]. A positive relationship was found between the interaction index and INSS and GQS (*p* < 0.001) (Table 3). This suggests that content that provides accurate and quality information attracts more attention. In addition, a negative relationship was observed between INSS and VIQI (*r* = −0.4006, *p* = 0.001), while a positive relationship was observed between INSS and m‐DISCERN and GQS (*p* < 0.05) (Table 3). Studies evaluating the content of YouTube videos on cancer and nutrition, as well as osteoporosis, have reported a positive correlation between GQS and m‐DISCERN in the former, and a very strong positive correlation between DISCERN and GQS in the latter [58, 59]. A separate study on complementary feeding reported a strong positive correlation between DISCERN and GQS scores [60], indicating that higher reliability of videos is associated with improved quality. This finding aligns with the results of our study and other similar research. The reason for both positive and negative correlations between INSS and the scales can be thought to be that INSS, m‐DISCERN and GQS use similar criteria in evaluating video quality, while VIQI makes a broader evaluation by taking into account visual and auditory elements such as aesthetics and narrative style.

In the correlation analysis, all scales except JAMA showed positive correlation among themselves (*p* < 0.05) (Table 3). The reason why JAMA did not show a correlation may be because JAMA is not a specific evaluation tool for YouTube videos but is used to evaluate information obtained from websites [61]. The use of this tool on YouTube is explained by reasons such as the need for users to access accurate information and to prevent misleading information in the field of health [62].

Evaluating YouTube videos as a source of information has been the subject of many studies recently [63, 64, 65]. Although studies evaluating TikTok videos on IBS are available in the literature [66, 67], we identified only one study [68] assessing YouTube videos. Hence, our study represents the second investigation of its kind.

Although this study focused on Turkish‐language videos, it has international relevance because YouTube is a global platform and its algorithm promotes content beyond national and language borders. Since social media use is high in Turkey and interest in online health information is increasing, findings from Turkish videos may help understand digital health communication in other non‐English‐speaking and middle‐income countries.

The current study also has certain limitations. First, only Turkish videos were included in the study, so our results cannot be generalised to other languages. Second, because of YouTube is a platform where new videos are constantly uploaded, our study findings may change over time.

## 5 Conclusion

Nutrition‐related video content has become a widely used source of information in recent years. While these resources can be beneficial, they also pose a risk of spreading misinformation. This study evaluated the reliability, quality, and user engagement metrics of YouTube videos related to nutrition recommendations for individuals with IBS. Despite the widespread reach of videos uploaded by non‐professionals, these videos often lacked evidence‐based content, as reflected by their lower m‐DISCERN, GQS, and VIQI scores compared to those uploaded by dietitians and gastroenterologists. However, interaction metrics such as the interaction index and VPI were significantly higher for videos uploaded by non‐professionals, indicating a potential gap between content popularity and scientific reliability.

These findings suggest that users often prioritise popularity and interactivity over quality of the source when selecting informational content. Additionally, the YouTube algorithm exposes users to popular content by highlighting popular videos. This underscores the need for healthcare professionals to adopt strategies that enhance the appeal and reach of their content, ensuring that accurate and reliable information can effectively compete in the digital space.

## Author Contributions

Conceptualization: Eda Başmısırlı and Merve Kip. Methodology: Eda Başmısırlı and Merve Kip. Data curation: Merve Kip and Hande Altun. Writing – original draft preparation: Eda Başmısırlı, Merve Kip, Hande Altun, and Neriman İnanç. Writing – review and editing: Eda Başmısırlı, Merve Kip, Hande Altun, and Neriman İnanç. Authors have read and agreed to the published version of the manuscript.

## Ethics Statement

The authors have nothing to report.

## Conflicts of Interest

The authors declare no conflicts of interest.

## 1 Peer Review

The peer review history for this article is available at https://www.webofscience.com/api/gateway/wos/peer-review/10.1111/jhn.70088.

## Supporting information

YouTube Research Revision‐Supplementary Material for Review.

## Data Availability

The data that support the findings of this study are available from the corresponding author upon reasonable request.
