# Supplementary material for: Evaluating the Quality and Reliability of YouTube Videos Providing Nutritional Recommendations for Irritable Bowel Syndrome
Source: J Hum Nutr Diet. 2025 Jul 3;38(4):e70088. doi: 10.1111/jhn.70088 (PMC12231924; doi:10.1111/jhn.70088)
Supplement: Supplementary file 1 — YouTube Research Revision‐Supplementary Material for Review. [file JHN-38-0-s001.docx]

**Supplementary Table 1. Irritable Bowel Syndrome Nutrition Scoring System (INSS)**

| **Number** | **Checklist** |
| --- | --- |
| 1 | A low FODMAP diet is recommended. |
| 2 | The use of soluble fibers is recommended. |
| 3 | The use of peppermint is recommended. |
| 4 | The use of probiotics is recommended. |
| 5 | It is recommended to consume at least 12 glasses of water daily (preferably water or other caffeine-free beverages like herbal teas). |
| 6 | Limiting tea and coffee consumption to 2-3 cups per day is recommended. |
| 7 | It is suggested that limiting the intake of high-fiber foods may be beneficial (e.g., wholemeal or high-fiber flour and bread, high-bran cereals, and whole grains such as brown rice and wheat bran). |
| 8 | Eating regular meals and taking time to eat is recommended. |
| 9 | It is recommended to avoid skipping meals or having long intervals between meals. |
| 10 | Reducing the consumption of alcohol and/or carbonated beverages is recommended. |
| 11 | It is recommended to reduce the intake of "resistant starch" commonly found in foods (starch that resists digestion in the small intestine and reaches the colon intact). |
| 12 | Limit fresh fruit intake to 3 portions per day (one portion is approximately 80 grams). |
| 13 | It is recommended that people with IBS (especially diarrhea-predominant IBS) should avoid sorbitol, an artificial sweetener found in sugar-free desserts (including gum) and drinks. |
| 14 | Limiting the intake of spicy foods is recommended. |
| 15 | Limiting fat intake is recommended. |
| 16 | Restricting dairy and dairy products (low-lactose diet) is recommended. |
| 17 | For individuals with constipation-predominant IBS, a trial of up to 2 tablespoons of flaxseed daily for 3 months is recommended. |
| 18 | It is recommended that individuals experiencing bloating may find it beneficial to consume oats (such as oatmeal or oat-based breakfast cereals) and flaxseed (up to 1 tablespoon daily). |
| 19 | The use of aloe vera for IBS treatment is discouraged. |

**Supplementary Figure 1.** Irritabl Bowel Syndrome Nutition Scoring System (INSS) Repat Counts
